# Effect of Statins for Primary Prevention of Cardiovascular Disease According to the Fatty Liver Index

**DOI:** 10.1007/s44197-024-00205-9

**Published:** 2024-02-23

**Authors:** Joonsang Yoo, Jimin Jeon, Minyoul Baik, Jinkwon Kim

**Affiliations:** 1 Department of Neurology, Yongin Severance Hospital, Yonsei University College of Medicine, 363, Dongbaekjukjeon-daero, Giheung-gu, Yongin-si, 16995 Republic of Korea; 2 Institute for Innovation in Digital Healthcare, Yonsei University Health System, Seoul, Korea

**Keywords:** Nonalcoholic fatty liver disease, Stroke, Myocardial infarction, Statins

## Abstract

**Introduction:**

Nonalcoholic fatty liver disease (NAFLD) is associated with increased risk of cardiovascular disease (CVD). We investigated the primary preventive effect of statins on CVD according to the level of fatty liver index (FLI), which is a marker of NAFLD.

**Methods:**

We conducted a nested case–control study on the basis of a nationwide health screening cohort in Korea. The participants were divided into tertiles (T1, T2, and T3) according to their FLI score. Cases were defined as individuals who developed CVD (composite of myocardial infarction and stroke). Three controls were matched to each case and multivariable conditional logistic regression analysis was performed.

**Results:**

Within a cohort of 206,263 participants without prior CVD, 7044 individuals suffered the primary outcome. For the nested case–control study, we selected these 7044 cases along with their corresponding 20,641 matched controls. Individuals in the T3 tertiles of FLI had a higher risk of CVD than those in the T1 tertile [adjusted odds ratio (OR) 1.30; 95% confidence interval (CI) 1.20–1.40, *P* < 0.001]. In sub-analyses based on FLI tertiles, statin therapy was associated with a lower risk of CVD (adjusted OR 0.72; 95% CI 0.61–0.85, *P* < 0.001) in the T3 tertile but not in the T1 and T2 tertiles.

**Conclusions:**

Statin therapy was associated with a reduced risk of CVD in individuals with high FLI but not in those with low FLI. Further research is needed to determine the pathophysiologic mechanism between statin and NAFLD.

**Supplementary Information:**

The online version contains supplementary material available at 10.1007/s44197-024-00205-9.

## Introduction

Nonalcoholic fatty liver disease (NAFLD) is 15–30% in the adult population and is gradually increasing in line with the increase in the prevalence of obesity or diabetes [1, 2]. NAFLD is diagnosed by hepatic steatosis on imaging or histology [3], but the fatty liver index (FLI) could be used as a useful index of NAFLD [4]. Overwhelming evidence indicates that patients with NAFLD are at increased risk of cardiovascular disease (CVD) as well as liver injury [5]. CVD death is the leading cause of death in patients with NAFLD [6]. Consequently, there is a great demand for a cardiovascular preventive strategy for individuals with NAFLD.

Use of statin, lipid-lowering medication that decreases hepatic cholesterol biosynthesis, is a key cornerstone in the primary and secondary prevention of atherosclerotic CVD [7–10]. The long-term use of statins is generally safe even in patients with CVD and abnormal liver function and is effective in the secondary prevention of further CVD [11, 12]. Therefore, statins are considered an effective therapeutic option for potentially counteracting both the progression of liver damage and the development of CVD from NAFLD [13, 14]. However, data are limited on whether statin therapy is effective in primary cardiovascular prevention in NAFLD patients and whether the preventive effect of statins differs depending on the presence or degree of NAFLD in the general population. In the current study, we assessed the effect and potential interaction of statins on CVD risk according to the level of FLI, which is a marker of NAFLD.

## Materials and Methods

### Study Design

We conducted a nested case–control study on the basis of the National Health Insurance System-Health Screening Cohort (NHIS-HEALS) in Korea [15]. The NHIS provides complimentary standardized health screening every 2 years for Korean adults aged 40 or older. The NHIS-HEALS cohort consisted of 514,866 people and was a 10% random sample of participants aged 40 to 79 years who underwent a national health screening program [15]. The NHIS-HEALS database contains health claims data for hospital visits, diagnoses, procedures, and prescriptions of all participants. The diagnosis at each hospital visit was based on the International Classification of Diseases 10th revision (ICD-10). This study was approved by the Institutional Review Board of Yongin Severance Hospital (9-2022-0169), and the requirement for informed consent was waived because of the retrospective nature of the study and the use of fully anonymized data from NHIS-HEALS.

### Study Population

We identified subjects who participated in the national health screening program between 2009 and 2010 from NHIS-HEALS. The index date of each subject was defined as the date of the health screening in 2009–2010. We excluded participants who had a history of CVD (ischemic heart diseases: I20–I25; stroke: I60–I64 and I69; and those who checked the history of stroke in the questionnaire) or cardiovascular procedures (carotid artery stent, carotid endarterectomy, coronary stent insertion, and coronary artery bypass graft) before the index date. We also excluded patients with a history of liver disease (chronic viral hepatitis: B18; alcohol liver disease: K70; liver cirrhosis: K74.6; and malignant neoplasm of liver and intrahepatic bile duct: C22) and participants who were consuming ≥ 2 drinks of alcohol per week. Participants with missing values for covariates or for whom FLI could not be calculated were excluded. Participants with a follow-up period of less than 30 days after the index date were excluded.

### Study Outcome

In the cohort, the primary outcome was defined as a composite development of myocardial infarction (MI) and stroke. MI was defined as an admission with a primary diagnosis of ICD-10 code I21. Stroke was defined as an admission with a primary diagnosis of ICD-10 code I60–I63 and was confirmed by performing brain computed tomography or magnetic resonance imaging during the admission period. The diagnostic accuracy of stroke and MI in the Korean national health claims database has been validated in previous studies [16, 17]. Study participants were followed up until the primary outcome; loss of participant eligibility; death; or December 31, 2019, whichever came first. If an outcome occurred multiple times during the follow-up period, the first event was considered the outcome. Secondary outcomes were defined as ischemic stroke (I63), hemorrhagic stroke (I60–I62), and MI, which are components of the primary outcome.

### Selection of Case and Control for the Nested Case–Control Study

In the nested case–control study, we set the case as a patient who suffered a primary outcome during the follow-up period. Using 1:3 incidence density sampling, we sampled three controls for each case with a replacement from all participants who were event free and at risk at the time of case occurrence. To minimize confounding effects attributable to differences in participant characteristics, each case was matched to its corresponding controls based on identical values of the following variables: sex, age (± 1 year is allowed), smoking status, alcohol consumption, physical activity, presence of diabetes mellitus, premorbid use of antiplatelets, and treatment with antiplatelets at the time of event occurrence. This process ensured an exact matching for these variables between each case and its corresponding controls.

### Assessment of Statin and Antiplatelet

The NHIS-HEALS database provided the prescription data of the statins and oral antiplatelets used by the study participants. Considering that medication administration typically changes over time, we took into account the time-varying feature of medication use during the follow-up period. In this nested case–control study, treatment with the medication was determined by exposure to the medication within the last 7 days at the time of event occurrence in the cases and at the corresponding time in the controls on the basis of the prescription records. The premorbid use of antiplatelets and statins were determined by exposure to the medications within seven days prior to the index date of health screening.

### Covariates

From the baseline health screening conducted in 2009–2010, we collected demographic data (sex, age, and quartile of household income), laboratory test results, comorbidities (hypertension, diabetes mellitus, renal disease, and atrial fibrillation), physical measurements (height, weight, and waist circumference), and questionnaires for lifestyle (smoking status, frequency of alcohol consumption per week, and physical activity) of the participants at the index date. Estimated glomerular filtration rate (eGFR) value was calculated according to the Modification of Diet in Renal Disease equation as follows: eGFR (mL/min per 1.73 m^2^) = 186 × Serum Cr(mg/dl)^−1.154^ × age^−0.203^ × 1.212 (if patient is black) × 0.742 (if female) [18]. Renal disease was defined as eGFR < 60 mL/min/1.73 m^2^. The presence of hypertension was determined if the patients received anti-hypertensive medications with corresponding diagnostic codes (ICD-10 code of I10–I13 or I15) or blood pressure ≥ 140/90 mmHg or if there was a positive check for hypertension in the self-report questionnaire. The presence of diabetes mellitus was determined if the patients received anti-diabetic medications with the related codes (E08–E11 or E13–E14), had a fasting glucose of > 7.0 mmol/L, or provided a positive check for diabetes in the self-report questionnaire. Atrial fibrillation was identified by the corresponding diagnostic code of I48. Smoking status was categorized into three groups: never smoker, former smoker, and current smoker. Physical activity was categorized into four groups on the basis of the metabolic equivalent of task (MET) minutes per week value calculated from the questionnaire (0, 1–499, 500–999, ≥ 1000). FLI was calculated using the measures in baseline health examination via the following formula [19]:

$${\text{FLI}} = \left( {{\text{e}}^{{0.{953}}} \times {\text{ln}}\left( {{\text{triglycerides}}} \right) + 0.{139} \times {\text{body mass index }}\left[ {{\text{BMI}}} \right] + 0.{718} \times {\text{ln}}\left( {\gamma - gt \, \left[ {{\text{gamma}} - {\text{glutamyl transferase}}} \right]} \right) + 0.0{53} \times {\text{waist circumference}} - {15}.{745}} \right)/\left( {{1} + {\text{e}}^{{0.{953}}} \times {\text{ln}}\left( {{\text{triglycerides}}} \right) + 0.{139 } \times {\text{BMI}} + 0.{718} \times {\text{ln}}\left( {\gamma - gt} \right) + 0.0{53} \times {\text{waist circumference}} - {15}.{745}} \right) \times {1}00.$$

### Statistical Analysis

Patient characteristics are expressed as number (%) for categorical variables and mean ± standard deviation for continuous variables. The normality of continuous variables was assessed using the Kolmogorov–Smirnov test. We performed multivariable conditional logistic regression with the matched case–control groups to calculate adjusted odds ratio (OR) and 95% confidence interval (CI). Adjustments were performed for household income, hypertension, atrial fibrillation, renal disease, premorbid use of statin, treatment with statin, and FLI. All statistical analyses were performed using SAS (version 9.4) and R software (version 4.3.0). All *P-*values were two-sided, and *P* < 0.05 was considered statistically significant.

## Results

### Characteristics of the Study Cohort

**Fig. 1:**
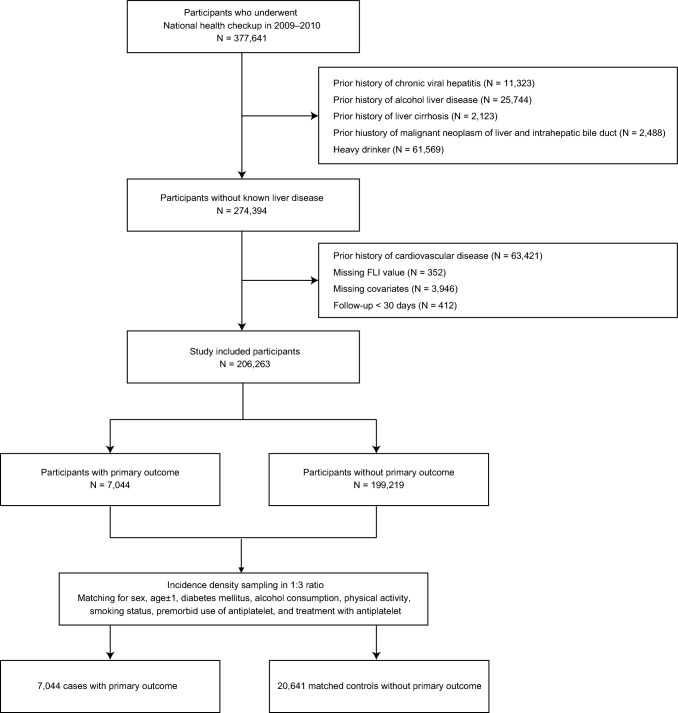
Flowchart for the selection of study participants in a nested case–control study for cardiovascular disease. *FLI* fatty liver index

According to the inclusion and exclusion criteria, 206,263 participants were selected and followed for the development of primary outcome (Fig. 1). In the cohort (Supplementary Table 1), there were 82,493 males (39.99%), and the mean age of the included participants was 58.5 ± 8.6 years. The mean of the FLI value was 25.06 ± 20.20. The cutoff value of T1, T2, T3 tertile of FLI was < 11.86, 11.86–29.14, and > 29.14, respectively. There were 13,316 patients (6.5%) who had premorbid statin use. The results of the Kolmogorov–Smirnov test, conducted to assess the normality of continuous variables, are presented in Supplementary Table 2.

### CVD Risk According to the FLI

During the mean follow-up period of 9.7 ± 1.6 years, 7044 participants suffered primary outcome (3.4%). In the nested case–control study (Table 1), we selected the 7044 cases with a primary outcome, and 20,641 corresponding controls. Compared with the T1 of FLI, the risk of the primary outcome was increased in the T2 (adjusted OR 1.09, 95% CI 1.01–1.17, *P* = 0.024) and T3 (adjusted OR 1.30, 95% CI 1.20–1.40,* P* < 0.001) of FLI. Treatment with statin was associated with decreased risk of primary outcome (adjusted OR 0.83, 95% CI 0.74–0.92, *P* < 0.001).

**Table 1:** Characteristics of the matched cases and controls

Variable	Case *N* = 7044	Control *N* = 20,641	Adjusted OR^a^ [95% CI]	*P*
Sex, male	3629 (51.52)	10,581 (51.26)	Matched	–
Age, years	64.68 ± 9.30	64.44 ± 9.19	Matched	–
Household income				
Q1, lowest	2155 (30.59)	6175 (29.92)	1 (ref)	
Q2	1798 (25.53)	5090 (24.66)	1.02 [0.95–1.10]	0.602
Q3	1891 (26.85)	5397 (26.15)	1.01 [0.94–1.09]	0.699
Q4, highest	1200 (17.04)	3979 (19.28)	0.87 [0.80–0.95]	< 0.001
Smoking status			Matched	–
Never smoker	4115 (58.42)	12,174 (58.98)		
Former smoker	1655 (23.50)	4834 (23.42)		
Current smoker	1274 (18.09)	3633 (17.60)		
Alcohol consumption, frequency per week			Matched	–
< 1 time	5796 (82.28)	17,138 (83.03)		
1 time	1248 (17.72)	3503 (16.97)		
Physical activity, MET-min/wk			Matched	–
0	2180 (30.95)	6361 (30.82)		
1–499	1906 (27.06)	5583 (27.05)		
500–999	1752 (24.87)	5150 (24.95)		
≥ 1000	1206 (17.12)	3547 (17.18)		
Hypertension	4038 (57.33)	10,229 (49.56)	1.37 [1.29–1.46]	< 0.001
Diabetes mellitus	1439 (20.43)	4054 (19.64)	Matched	–
Atrial fibrillation	239 (3.39)	385 (1.87)	1.76 [1.49–2.08]	< 0.001
Renal disease	800 (11.36)	2017 (9.77)	1.13 [1.03–1.23]	0.011
Premorbid medication				
Statin	516 (7.33)	1648 (7.98)	0.87 [0.78–0.97]	0.016
Antiplatelet	995 (14.13)	2729 (13.22)	Matched	–
Treatment				
Statin	600 (8.52)	1936 (9.38)	0.83 [0.74–0.92]	< 0.001
Antiplatelet	806 (11.44)	2223 (10.77)	Matched	–
Fatty liver index	30.03 ± 21.30	27.39 ± 20.50		
T1 (< 11.856)	1635 (23.21)	5611 (27.18)	1 (ref)	–
T2 (11.856–29.138)	2337 (33.18)	7200 (34.88)	1.09 [1.01–1.17]	0.024
T3 (> 29.138)	3072 (43.61)	7830 (37.93)	1.30 [1.20–1.40]	< 0.001

*CI* confidence interval, *MET-min/wk* metabolic equivalent of task-minutes per week, *OR* odds ratio, *Q* quartile, *T* tertile

^a^Adjustments were made to household income, renal disease, hypertension, atrial fibrillation, and premorbid use of statin, treatment with statin, and fatty liver index

### Effect of Statins on CVD According to the FLI

**Fig. 2:**
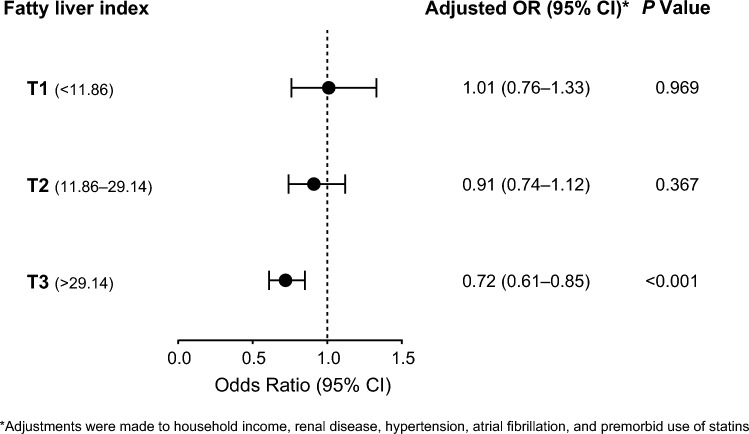
Effect of statin treatment on the primary outcome according to the fatty liver index. The data were obtained from nested case–control studies for the primary outcome conducted with the three sub-cohorts on the basis of the level of fatty liver index (T1, T2, T3). *CI* confidence interval, *OR* odds ratio

When we conducted nested case–control studies involving sub-populations of T1, T2, and T3 tertiles of FLI (Fig. 2), we found that statin use in the T1 (adjusted OR 1.01, 95% CI 0.76–1.33, *P* = 0.969) and T2 tertiles (adjusted OR 0.91, 95% CI 0.74–1.12, *P* = 0.367) were not related to the occurrence of the primary outcome. However, statin use in the T3 tertile was associated with a 25% lower risk of cardiovascular outcome (adjusted OR 0.72, 95% CI 0.61–0.85, *P* < 0.001).

### Analysis in T3 Tertile of FLI

**Fig. 3:**
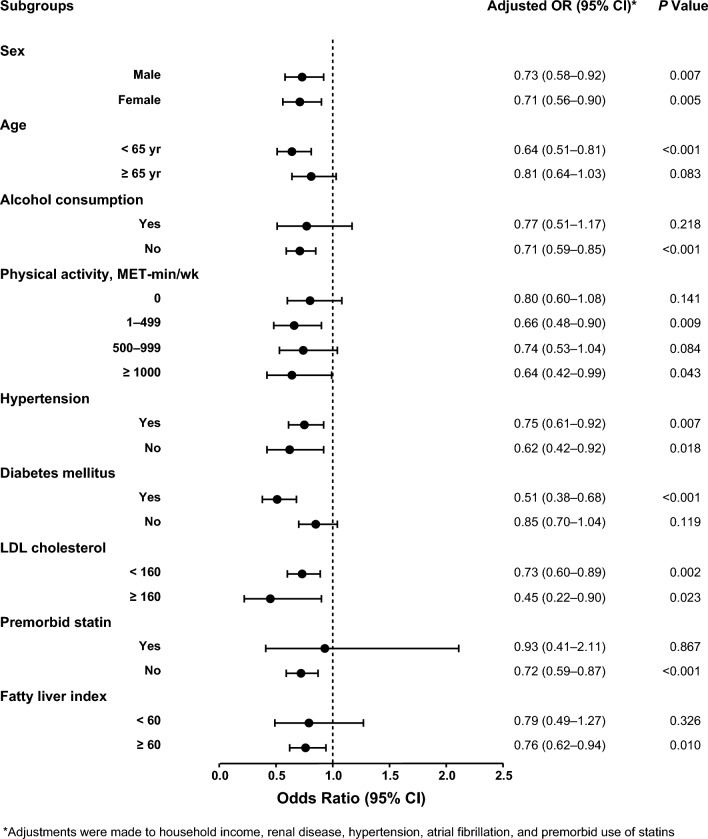
Subgroup analyses for the effect of statins on the primary outcome in a nested case–control study based on the third tertile of the fatty liver index*. MET-min/wk* metabolic equivalent of task-minutes per week, *LDL* low-density lipoprotein, *CI* confidence interval, *OR* odds ratio

Supplementary Table 3 shows the result of the nested case–control study conducted with the individuals in the T3 tertile of FLI (3072 cases and 8771 controls). When we performed subgroup analysis on the basis of covariates (Fig. 3), the use of statin was consistently associated with a decreased risk of primary outcome. In the secondary outcome analyses with the T3 of FLI (Table 2), statin treatment exhibited a potential to reduce the risk of ischemic stroke, hemorrhagic stroke, and MI. However, only the reduction in the risk of ischemic stroke (adjusted OR 0.61, 95% CI 0.49–0.77) reached statistical significance.

**Table 2:** Risk for individual outcome according to statin treatment in the third tertile of fatty liver index

Outcome	Adjusted OR [95% CI]^a^		
	Hemorrhagic stroke (*N* = 440)	Ischemic stroke (*N* = 1711)	Myocardial infarction (*N* = 921)
Statin	0.85 [0.56–1.29]	0.61 [0.49–0.77]	0.86 [0.64–1.16]

The data are obtained from multivariable conditional logistic regression analyses using the case–control dataset matched for sex, age, diabetes mellitus, alcohol consumption, physical activity, smoking status, premorbid use of antiplatelets, and treatment with antiplatelets

*CI* confidence interval, *OR* odds ratio

^a^Adjustments were made to household income, hypertension, atrial fibrillation, renal disease, and premorbid use of statins

### Sensitivity Analysis

Because the commonly used criterion for FLI in identifying NAFLD is ≥ 60, we evaluated the association between statin therapy and the risk of CVD in subpopulations with FLI ≥ 60. Among those with FLI ≥ 60, the use of statins was also associated with a reduced occurrence of CVD (adjusted OR 0.67, 95% CI [0.48–0.93]) (Supplementary Table 4). As an additional sensitivity analysis, we validated the results of this study using the hepatic steatosis index (HSI), which is another simple screening tool that reflects NAFLD instead of FLI [20]. In our data, the cutoff value of T1, T2, T3 tertile of HSI was < 30.62, 30.62–34.28, and > 34.28, respectively (Supplementary Table 5). In the analyses with sub-cohorts based on T1, T2, and T3 of HSI, statin treatment was associated with lower occurrence of CVD (adjusted OR 0.73, 95% CI 0.61–0.87, *P* < 0.001) in the patients with T3 tertile of HSI, but not in T1 and T2 tertiles of HSI (Supplementary Fig. 1). The findings based on HSI were completely consistent with the results based on FLI.

## Discussion

This study investigated the relationship between FLI, which is a simple marker of NAFLD, and CVD risk in approximately 200,000 adults of the general population who participated in a national health screening. We explored the primary cardiovascular preventive effect of statins according to the level of FLI. Consistent with previous epidemiologic knowledge, CVD occurred frequently in participants with high FLI, and the use of statins was associated with a low incidence of CVD. However, we found that treatment with statins did not reduce the risk of CVD in participants with low FLI, whereas the reduction of CVD with statin treatment was only observed in participants with high FLI, thus indicating the presence of NAFLD. Among the participants with high FLI, the beneficial effect of statin was consistent regardless of premorbid statin use and comorbidities. The varying effect of statins according to the level of FLI was reaffirmed in the sensitivity analysis on the basis of HSI, which is another marker of NAFLD.

Patients with NAFLD are at increased risk for CVD and hepatic mortality and morbidity [21, 22]. In a recent meta-analysis of seven observational studies, NAFLD was reported as a pooled OR of 1.27 for fatal or nonfatal CVD [23]. In a large cohort of patients with histologically confirmed NAFLD, excess CVD risk was evident across all stages of NAFLD and increased with worsening severity of NAFLD [24]. Regarding the high CVD risk of patients with NAFLD, such patients are recommended to receive comprehensive workup and risk screening for CVD [13].

There are several putative mechanisms linking NAFLD and CVD [25]. At first, NAFLD and CVD share several cardiometabolic risk factors, such as dyslipidemia, metabolic syndrome, central obesity, sarcopenia, diabetes mellitus, hypertension, chronic kidney disease, and poor lifestyle choices [21, 26]. Furthermore, NAFLD might be an independent risk factor for CVD beyond the shared traditional risk factors [6, 21, 25]. There is a bi-directional relationship between the cardiometabolic risk factors and NAFLD. A study of 16,000 health screening subjects over a 10-year follow-up period reported that an increase in FLI was associated with a higher occurrence of hypertension [27]. Similarly, subjects with NAFLD are at an increased risk of developing diabetes mellitus and metabolic syndrome [28]. There is a notable positive association between NAFLD and systemic atherosclerosis, including coronary artery calcification, carotid artery atherosclerosis, and arterial stiffness [29, 30]. NAFLD severity is correlated with the severity of coronary artery disease and vulnerability of coronary plaque [31]. The development and progression of atherosclerosis in NAFLD may be driven by factors such as endothelial dysfunction, altered lipid metabolism, and systemic insulin resistance [26]. Beyond atherosclerosis, an increase in thrombogenic molecules and proinflammatory factors, as well as impaired fibrinolysis, in NAFLD leads to a procoagulant state, which is linked to the occurrence of CVD [32, 33].

Statins effectively decrease the LDL (low-density lipoprotein) cholesterol level, an important causal risk factor of atherosclerotic CVD. On the basis of evidence, practical guidelines against CVD strongly recommend statin therapy as the primary and secondary preventive therapy for high-risk individuals [7, 34]. Beyond the lipid-lowering effect, statins have multiple cardiovascular protective effects, such as improving endothelial dysfunction, increasing nitric oxide bioavailability, reducing inflammatory and oxidative stress, preventing thrombogenicity, enhancing fibrinolysis, inhibiting proliferation, stabilizing endothelium, and inducing immune modulation (i.e., pleiotropic effect) [35]. Owing to the anti-lipid and anti-inflammatory characteristics of statins, statin therapy may prevent progression to nonalcoholic steatohepatitis and fibrosis among patients with NAFLD [36]. In animal models, statins reduce NAFLD-associated hepatic lipotoxicity, oxidative stress, and inflammatory response via multiple pathways [35, 37]. According to the increasing knowledge on NAFLD and physiological mechanisms, statin therapy appears to be a viable treatment option for patients with NAFLD in terms of reducing inflammation, oxidative stress, and cardiovascular risk [38].

Interestingly, prior studies have suggested that the benefit of statins in reducing CVD risk is greater in patients with NAFLD than in those without NAFLD [11, 12]. Our findings align with previous studies, showing that statin use was associated with reduced CVD only in those with high FLI regardless of their LDL cholesterol level. These data suggest that statins have a multifaceted effect of reducing CVD beyond their LDL-lowering ability in patients with NAFLD. However, given the lack of significant reduction in CVD risk with statins in patients with low FLI, the use of statins for primary prevention should be personalized on the basis of individual risk assessments.

Our study supports the extensive use of statins in patients with NAFLD, including those without a history of CVD. On the basis of established evidence, the guidelines on CVD prevention recommend the administration of statin to patients with CVD or those who are at high risk of developing CVD; however, data are still limited on the use of statins as primary prevention for patients with NAFLD [14, 39]. In line with our findings, a post hoc analysis of the Assessing The Treatment Effect in Metabolic Syndrome Without Perceptible diabetes trial, which focused on the primary prevention of CVD in participants with NAFLD, revealed the lower occurrence of CVD in a group with more intensive atorvastatin therapy than a group with less intensive atorvastatin therapy [40]. Statins are still under-prescribed in patients with NAFLD who are considered high risk of CVD or who already meet the established criteria for statin therapy [41]. According to recent cohort studies, nearly half of patients with NAFLD are at intermediate to high risk of developing atherosclerotic CVD over a 10-year period, but the rate of statin use falls short of half of these patients, thus indicating a significant treatment gap [42, 43].

Concerns about statin hepatotoxicity often discourage the use of statins in patients with NAFLD, resulting in the underutilization of statins and missed opportunities for the prevention of CVD [44]. However, data have shown that statin therapy is relatively safe and effective approach for reducing inappropriate lipid accumulation, hepatic lipotoxicity, steatosis, and liver fibrosis in patients with NAFLD [45, 46]. In patients with abnormal liver tests and a history of coronary heart disease, the use of statins was safe and effective for the secondary prevention of CVD [11, 12]. Moreover, several studies have indicated that the appropriate use of statins is associated with a reduction in the risk of developing NAFLD, progression of nonalcoholic steatohepatitis, and development of hepatocellular carcinoma [47, 48].

This study has several limitations. First, this study was conducted retrospectively; therefore, it lacks the ability to establish causality. This study was also conducted in a homogeneous ethnic group of a single country, and caution is needed when generalizing the results. Due to the limitations of research using health insurance data, we did not have access to detailed information on clinical symptoms, specific laboratory data, indications for the statin prescription, and liver imaging or histologic studies, which are required to confirm a diagnosis of NAFLD. Lastly, it is unclear whether the benefits of statin therapy in the high FLI group are attributable to the established cardiovascular protective effect of statins or if they arise from direct improvements in NAFLD.

## Conclusions

In this population-based, nested case–control study, statin therapy was associated with a reduced risk of CVD in patients with high FLI, which is a marker of NAFLD, but not in those with low FLI. Additional research is needed to determine the pathophysiologic mechanism between statin and NAFLD and the pharmacological implications of statins to patients with NAFLD.

## Supplementary Information

Below is the link to the electronic supplementary material.

Supplementary file1 (DOCX 77 KB)

## Data Availability

The dataset supporting the findings of this study can be obtained from NHIS in Korea, but with restrictions to data availability. The use of the dataset is restricted to the current research under license; therefore, public access of the dataset is not available. Researchers are only access the data upon reasonable request with approval from the inquiry committee of research support in NHIS (https://nhiss.nhis.or.kr/bd/ab/bdaba032eng.do).

## Abbreviations

CI: Confidence interval
CVD: Cardiovascular disease
eGFR: Estimated glomerular filtration rate
FLI: Fatty liver index
HSI: Hepatic steatosis index
ICD-10: International Classification of Diseases 10th revision
LDL: Low-density lipoprotein
MET: Metabolic equivalent of task
MI: Myocardial infarction
NAFLD: Nonalcoholic fatty liver disease
NHIS-HEALS: National Health Insurance System-Health Screening Cohort
OR: Odds ratio
