# Supplementary material for: Effect of Statins for Primary Prevention of Cardiovascular Disease According to the Fatty Liver Index
Source: J Epidemiol Glob Health. 2024 Feb 23;14(3):710–9. doi: 10.1007/s44197-024-00205-9 (PMC11442725; doi:10.1007/s44197-024-00205-9)
Supplement: Supplementary file 1 — Supplementary file1 (DOCX 77 KB) [file 44197_2024_205_MOESM1_ESM.docx]

**SUPPLEMENTARY MATERIALS**

**Supplementary Figure.** Effect of statin treatment on the primary outcome according to the hepatic steatosis index


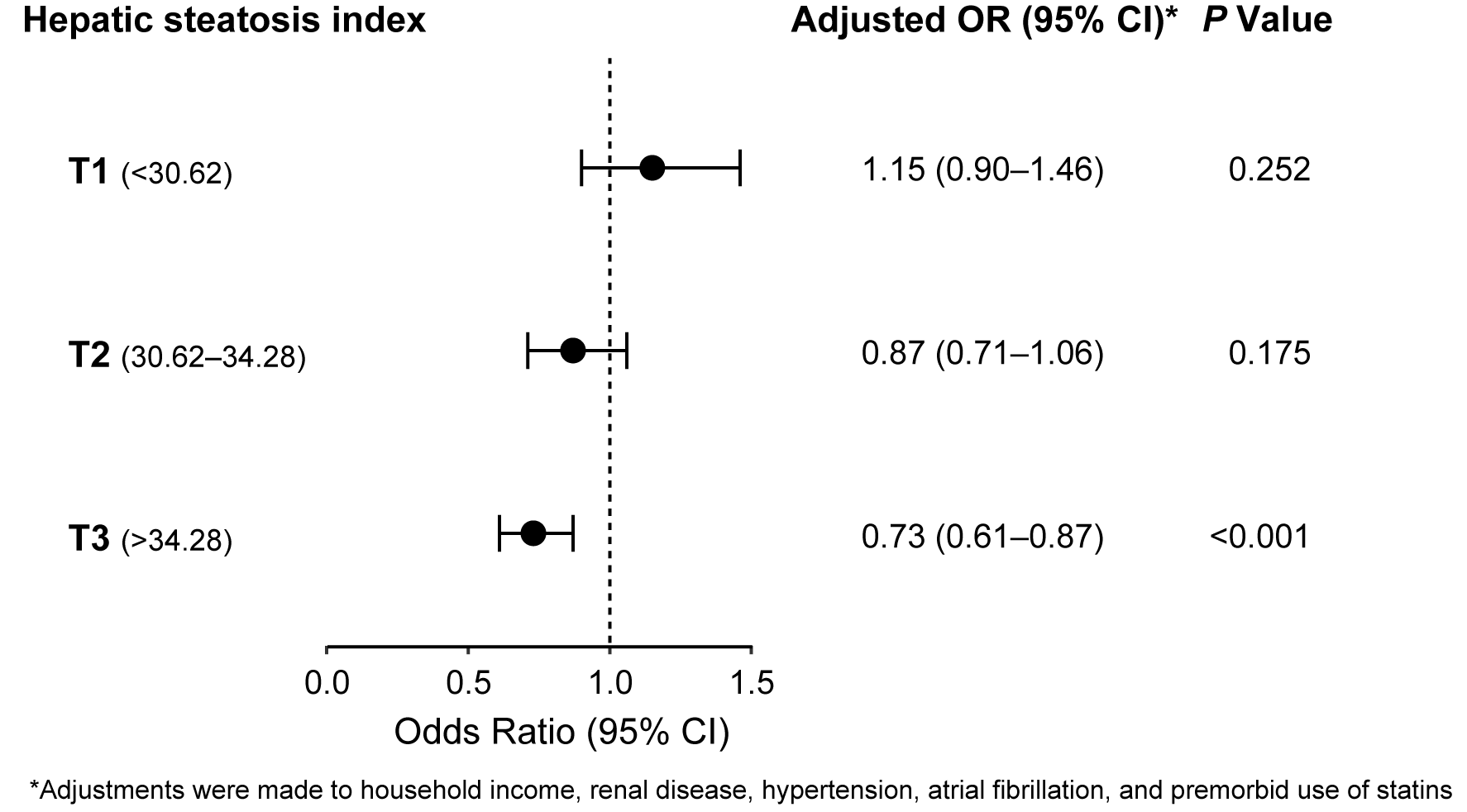


The data were obtained from nested case–control studies for the primary outcome conducted with the three sub-cohorts on the basis of the level of hepatic steatosis index (T1, T2, T3). *CI* confidence interval, *OR* odds ratio.

**Supplementary Table 1.** Baseline characteristics of the included patients

| Variable | Total (n = 206,263) | Fatty liver index | | |
| --- | --- | --- | --- | --- |
|  |  | T1 (<11.86)  (n = 68,754) | T2 (11.86–29.14)  (n = 68,755) | T3 (>29.14)  (n = 68,754) |
| Sex, male | 82,493 (39.99) | 17,428 (25.35) | 26,922 (39.16) | 38,143 (55.48) |
| Age, years | 58.50 ± 8.64 | 57.46 ± 8.72 | 59.09 ± 8.63 | 58.96 ± 8.46 |
| Household income |  |  |  |  |
| Q1, lowest | 59,135 (28.67) | 21,118 (30.72) | 19,417 (28.24) | 18,600 (27.05) |
| Q2 | 52,718 (25.56) | 17,277 (25.13) | 17,720 (25.77) | 17,721 (25.77) |
| Q3 | 54,874 (26.60) | 17,665 (25.69) | 18,256 (26.55) | 18,953 (27.57) |
| Q4, highest | 39,536 (19.17) | 12,694 (18.46) | 13,362 (19.43) | 13,480 (19.61) |
| Smoking status |  |  |  |  |
| Never smoker | 142,717 (69.19) | 55,373 (80.54) | 48,380 (70.37) | 38,964 (56.67) |
| Former smoker | 40,812 (19.79) | 8,455 (12.30) | 13,266 (19.29) | 19,091 (27.77) |
| Current smoker | 22,734 (11.02) | 4,926 (7.16) | 7,109 (10.34) | 10,699 (15.56) |
| Alcohol consumption, frequency per week |  |  |  |  |
| <1 time | 163,917 (79.47) | 57,942 (84.27) | 55,227 (80.32) | 50,748 (73.81) |
| 1 time | 42,346 (20.53) | 10,812 (15.73) | 13,528 (19.68) | 18,006 (26.19) |
| Physical activity, MET-min/wk |  |  |  |  |
| 0 | 54,942 (26.64) | 17,860 (25.98) | 18,192 (26.46) | 18,890 (27.47) |
| 1–499 | 59,127 (28.67) | 19,648 (28.58) | 19,336 (28.12) | 20,143 (29.30) |
| 500–999 | 53,395 (25.89) | 18,116 (26.35) | 17,930 (26.08) | 17,349 (25.23) |
| ≥1,000 | 38,799 (18.81) | 13,130 (19.10) | 13,297 (19.34) | 12,372 (17.99) |
| BMI, kg/m^2^ | 23.85 ± 2.92 | 21.48 ± 1.99 | 23.82 ± 1.92 | 26.25 ± 2.55 |
| Waist circumference, cm | 80.65 ± 8.20 | 73.29 ± 5.51 | 80.77 ± 5.07 | 87.89 ± 6.27 |
| Laboratory findings |  |  |  |  |
| Fasting glucose, mmol/L | 5.48 ± 1.27 | 5.21 ± 0.97 | 5.44 ± 1.18 | 5.80 ± 1.53 |
| Total cholesterol, mmol/L | 5.24 ± 0.96 | 5.05 ± 0.89 | 5.26 ± 0.94 | 5.43 ± 1.00 |
| LDL cholesterol, mmol/L | 3.17 ± 0.95 | 3.08 ± 0.88 | 3.24 ± 0.94 | 3.19 ± 1.02 |
| HDL cholesterol, mmol/L | 1.42 ± 0.68 | 1.54 ± 0.57 | 1.40 ± 0.68 | 1.31 ± 0.77 |
| Triglyceride, mmol/L | 1.48 ± 0.90 | 0.94 ± 0.38 | 1.39 ± 0.58 | 2.12 ± 1.12 |
| γ-GT, U/L | 27.80 ± 29.54 | 16.67 ± 7.96 | 23.77 ± 15.39 | 42.96 ± 44.14 |
| Comorbidity |  |  |  |  |
| Hypertension | 78,839 (38.22) | 17,099 (24.87) | 26,718 (38.86) | 35,022 (50.94) |
| Diabetes mellitus | 24,095 (11.68) | 4,089 (5.95) | 7,486 (10.89) | 12,520 (18.21) |
| Atrial fibrillation | 2,886 (1.40) | 768 (1.12) | 993 (1.44) | 1,125 (1.64) |
| Renal disease | 13,835 (6.71) | 3,550 (5.16) | 4,708 (6.85) | 5,577 (8.11) |
| Premorbid medication |  |  |  |  |
| Statin | 13,316 (6.45) | 2,576 (3.75) | 4,565 (6.64) | 6,175 (8.98) |
| Antiplatelet | 18,752 (9.09) | 3,378 (4.91) | 6,276 (9.13) | 9,098 (13.23) |

*MET-min/wk* metabolic equivalent of task-minutes per week, *BMI* body mass index, *LDL* low-density lipoprotein, *HDL* high-density lipoprotein, *Q* quartile, *T* tertile, *γ-GT* gamma-glutamyl transferase.

The data are represented as number (%) or mean ± standard deviation.

**Supplementary Table 2.** The results of the Kolmogorov-Smirnov test for continuous variable in the entire cohort (N=206,263)

| Variable |  | **Kolmogorov-Smirnov Test** | |
| --- | --- | --- | --- |
|  | mean ± standard deviation | *D* | p-value |
| Age, years | 58.50 ± 8.64 | 1.000 | <0.001 |
| BMI, kg/m^2^ | 23.85 ± 2.92 | 1.000 | <0.001 |
| Waist circumference, cm | 80.65 ± 8.20 | 0.9999 | <0.001 |
| Laboratory findings |  |  |  |
| Fasting glucose, mmol/L | 5.48 ± 1.27 | 0.9993 | <0.001 |
| Total cholesterol, mmol/L | 5.24 ± 0.96 | 0.9962 | <0.001 |
| LDL cholesterol, mmol/L | 3.17 ± 0.95 | 0.9218 | <0.001 |
| HDL cholesterol, mmol/L | 1.42 ± 0.68 | 0.7779 | <0.001 |
| Triglyceride, mmol/L | 1.48 ± 0.90 | 0.6670 | <0.001 |
| γ-GT, U/L | 27.80 ± 29.54 | 0.9996 | <0.001 |
| Fatty liver index | 25.06 ± 20.20 | 0.9669 | <0.001 |

*D* (D statistic) and p-value are derived from the Kolmogorov-Smirnov test.

**Supplementary Table 3.** Characteristics of the matched cases and controls in T3 tertile of fatty liver index

| Variable | Case  N = 3,072 | Control  N = 8,771 | Adjusted OR^a^  [95% CI] | P value |
| --- | --- | --- | --- | --- |
| Sex, male | 1,904 (61.98) | 5,418 (61.77) | Matched | - |
| Age, years | 63.58 ± 9.08 | 63.16 ± 8.91 | Matched | - |
| Household income |  |  |  |  |
| Q1, lowest | 930 (30.27) | 2,498 (28.48) | 1 (ref) |  |
| Q2 | 780 (25.39) | 2,200 (25.08) | 0.96 [0.86–1.08] | 0.491 |
| Q3 | 827 (26.92) | 2,366 (26.98) | 0.95 [0.85–1.06] | 0.375 |
| Q4, highest | 535 (17.42) | 1,707 (19.46) | 0.84 [0.74–0.96] | 0.009 |
| Smoking status |  |  | Matched | - |
| Never smoker | 1,548 (50.39) | 4,464 (50.89) |  |  |
| Former smoker | 889 (28.94) | 2,550 (29.07) |  |  |
| Current smoker | 635 (20.67) | 1,757 (20.03) |  |  |
| Alcohol consumption,  frequency per week |  |  | Matched | - |
| <1 time | 2,403 (78.22) | 6,971 (79.48) |  |  |
| 1 time | 669 (21.78) | 1,800 (20.52) |  |  |
| Physical activity, MET-min/wk |  |  | Matched | - |
| 0 | 918 (29.88) | 2,601 (29.65) |  |  |
| 1-499 | 862 (28.06) | 2,477 (28.24) |  |  |
| 500-999 | 785 (25.55) | 2,243 (25.57) |  |  |
| ≥1000 | 507 (16.50) | 1,450 (16.53) |  |  |
| Hypertension | 1,955 (63.64) | 5,057 (57.66) | 1.29 [1.18–1.42] | <0.001 |
| Diabetes mellitus | 804 (26.17) | 2,161 (24.64) | Matched | - |
| Atrial fibrillation | 99 (3.22) | 159 (1.81) | 1.77 [1.37–2.30] | <0.001 |
| Renal disease | 380 (12.37) | 920 (10.49) | 1.15 [1.00–1.31] | 0.047 |
| Premorbid medication |  |  |  |  |
| Statin | 262 (8.53) | 905 (10.32) | 0.78 [0.67–0.92] | 0.003 |
| Antiplatelet | 528 (17.19) | 1,349 (15.38) | Matched | - |
| Treatment |  |  |  |  |
| Statin | 280 (9.11) | 980 (11.17) | 0.72 [0.61–0.85] | <0.001 |
| Antiplatelet | 387 (12.60) | 1,002 (11.42) | Matched | - |

^a^Adjustments were made to household income, hypertension, atrial fibrillation, renal disease, and premorbid use of statins.

*CI* confidence interval, *MET-min/wk* metabolic equivalent of task-minutes per week, *OR* odds ratio, *Q* quartile, *T* tertile.

**Supplementary Table 4.** Effect of statins on the primary outcome in a nested case–control study within the subpopulations with fatty liver index ≥60

| Variable | Case  N = 774 | Control  N = 2,000 | Adjusted OR^a^  [95% CI] | P value |
| --- | --- | --- | --- | --- |
| Sex, male | 527 (68.09) | 1351 (67.55) | Matched |  |
| Age, years | 61.98 ± 9.35 | 60.76 ± 8.88 | Matched |  |
| Household income |  |  |  |  |
| Q1, lowest | 239 (30.88) | 578 (28.90) | 1 (ref) |  |
| Q2 | 212 (27.39) | 497 (24.85) | 1.08 [0.85–1.36] | 0.526 |
| Q3 | 199 (25.71) | 559 (27.95) | 0.85 [0.67–1.07] | 0.169 |
| Q4, highest | 124 (16.02) | 366 (18.30) | 0.80 [0.61–1.05] | 0.110 |
| Smoking status |  |  | Matched |  |
| Never smoker | 328 (42.38) | 857 (42.85) |  |  |
| Former smoker | 269 (34.75) | 692 (34.60) |  |  |
| Current smoker | 177 (22.87) | 451 (22.55) |  |  |
| Alcohol consumption,  frequency per week |  |  | Matched |  |
| <1 time | 572 (73.90) | 1500 (75.00) |  |  |
| 1 time | 202 (26.10) | 500 (25.00) |  |  |
| Physical activity, MET-min/wk |  |  | Matched |  |
| 0 | 228 (29.46) | 584 (29.20) |  |  |
| 1–499 | 234 (30.23) | 611 (30.55) |  |  |
| 500–999 | 203 (26.23) | 519 (25.95) |  |  |
| ≥1000 | 109 (14.08) | 286 (14.30) |  |  |
| Hypertension | 512 (66.15) | 1222 (61.10) | 1.10 [0.90–1.35] | 0.357 |
| Diabetes mellitus | 237 (30.62) | 555 (27.75) | Matched |  |
| Atrial fibrillation | 32 (4.13) | 35 (1.75) | 2.12 [1.26–3.57] | 0.005 |
| Renal disease | 103 (13.31) | 172 (8.60) | 1.39 [1.04–1.85] | 0.027 |
| Premorbid medication |  |  |  |  |
| Statin | 76 (9.82) | 202 (10.10) | 0.90 [0.65–1.23] | 0.507 |
| Antiplatelet | 146 (18.86) | 280 (14.60) | Matched |  |
| Treatment |  |  |  |  |
| Statin | 78 (10.08) | 248 (12.40) | 0.67 [0.48–0.93] | 0.017 |
| Antiplatelet | 98 (12.66) | 187 (9.35) | Matched |  |

^a^Adjustments were made to household income, renal disease, hypertension, atrial fibrillation, and premorbid use of statin, treatment with statin, and fatty liver index.

*CI* confidence interval, *MET-min/wk* metabolic equivalent of task-minutes per week, *OR* odds ratio, *Q* quartile, *T* tertile

**Supplementary Table 5.** Baseline characteristics of the included patients according to tertiles of hepatic steatosis index

| Variable | Hepatic steatosis index | | |
| --- | --- | --- | --- |
|  | T1 (<30.62)  (n = 68,755) | T2 (30.62–34.28)  (n = 68,754) | T3 (>34.28)  (n = 68,754) |
| Sex, male | 30,960 (45.03) | 25,827 (37.56) | 25,706 (37.39) |
| Age, years | 58.88 ± 9.36 | 58.34 ± 8.42 | 58.28 ± 8.07 |
| Hepatic steatosis index | 28.15 ± 1.93 | 32.40 ± 1.04 | 37.78 ±3.44 |
| Household income |  |  |  |
| Q1, lowest | 19,961 (29.03) | 19,640 (28.57) | 19,534 (28.41) |
| Q2 | 17,263 (25.11) | 17,320 (25.19) | 18,135 (26.38) |
| Q3 | 18,010(26.19) | 18,263 (26.56) | 18,601 (27.05) |
| Q4, highest | 13,521 (19.67) | 13,531 (19.68) | 12,484 (18.16) |
| Smoking status |  |  |  |
| Never smoker | 45,466 (66.13) | 48,992 (71.26) | 48,259 (70.19) |
| Former smoker | 14,262 (20.74) | 13,186 (19.18) | 13,364 (19.44) |
| Current smoker | 9,027 (13.13) | 6,576 (9.56) | 7,131 (10.37) |
| Alcohol consumption, frequency per week |  |  |  |
| <1 time | 54,066 (78.64) | 54,547 (79.34) | 55,304 (80.44) |
| 1 time | 14,689 (21.36) | 14,207 (20.66) | 13,450 (19.56) |
| Physical activity, MET-min/wk |  |  |  |
| 0 | 18,173 (26.43) | 17,622 (25.63) | 19,147 (27.85) |
| 1-499 | 19,629 (28.55) | 19,584 (28.48) | 19,914 (28.96) |
| 500-999 | 18,027 (26.22) | 18,012 (26.20) | 17,356 (25.24) |
| ≥1,000 | 12,926 (18.80) | 13,536 (19.69) | 12,337 (17.94) |
| BMI, kg/m^2^ | 21.26 ± 1.79 | 23.83 ± 1.59 | 26.46 ± 2.49 |
| Waist circumference, cm | 75.26 ± 6.80 | 80.53 ± 6.56 | 86.17 ± 7.29 |
| Laboratory findings |  |  |  |
| Fasting glucose, mmol/L | 5.20 ± 0.86 | 5.40 ± 1.13 | 5.85 ± 1.61 |
| Total cholesterol, mmol/L | 5.11 ± 0.91 | 5.27 ± 0.95 | 5.35 ± 1.01 |
| LDL cholesterol, mmol/L | 3.09 ± 0.89 | 3.20 ± 0.96 | 3.22 ± 1.00 |
| HDL cholesterol, mmol/L | 1.50 ± 0.75 | 1.42 ± 0.68 | 1.35 ± 0.61 |
| Triglyceride, mmol/L | 1.23 ± 0.73 | 1.48 ± 0.87 | 1.74 ± 1.02 |
| γ-GT, U/L | 22.06 ± 23.50 | 25.97 ± 26.21 | 35.36 ± 35.86 |
| Comorbidity |  |  |  |
| Hypertension | 19,307 (28.08) | 25,399 (36.94) | 34,133 (49.65) |
| Diabetes mellitus | 2,555 (3.72) | 6,166 (8.97) | 15,374 (22.36) |
| Atrial fibrillation | 946 (1.38) | 942 (1.37) | 998 (1.45) |
| Renal disease | 4,374 (6.36) | 4,605 (6.70) | 4,856 (7.06) |
| Pre-morbid medication |  |  |  |
| Statin | 1,992 (2.90) | 4,091 (5.95) | 7,233 (10.52) |
| Antiplatelet | 3,612 (5.25) | 5,794 (8.43) | 9,346 (13.59) |
| Fatty liver index |  |  |  |
| T1 (<11.86) | 45,347 (65.95) | 19,458 (28.30) | 3,949 (5.74) |
| T2 (11.86–29.14) | 18,727 (27.24) | 30,777 (44.76) | 19,251 (28.00) |
| T3 (>29.14) | 4,681 (6.81) | 18,519 (26.94) | 45,554 (66.26) |

The data are represented as numbers (%) or means ± standard deviation.

*MET-min/wk* metabolic equivalent of task-minutes per week, *BMI* body mass index, *LDL* low-density lipoprotein, *HDL* high-density lipoprotein, *Q* quartile, *T* tertile, *γ-GT* gamma-glutamyl transferase.
